# MULTIDIMENSIONAL FRAILTY SCORE IS SUPERIOR TO PREDICT COMPLICATIONS AFTER SURGERY THAN CONVENTIONAL RISK FACTORS

**DOI:** 10.1093/geroni/igz038.2531

**Published:** 2019-11-08

**Authors:** Jung-Yeon Choi, Kwang-il Kim, Hee-won Jung, Cheol-Ho Kim, Sung-Bum Kang, Ho-Seong Han, HyungHo Kim

**Affiliations:** Seoul National Bundang Hospital, Bundang-gu, Seongnam-si, Korea, Republic of

## Abstract

Frail older adults are at increased risk for postoperative morbidity compared with their robust counterparts. We compared predictive utility of multidimensional frailty score (MFS) with physical performance parameters or conventional risk stratification indicators to identify postoperative complication in older surgical patients. From January 2016 to June 2017, 648 older surgical patients (age≥ 65) were included for analysis. The MFS was calculated through comprehensive geriatric assessment (CGA). Grip strength and gait speed were measured preoperatively. The primary outcome was postoperative complication (eg, pneumonia, urinary tract infection, delirium, acute pulmonary thromboembolism, and unplanned ICU admission). Secondary outcome was 6-months all-cause mortality. Sixty-six (10.2%) patients experienced postoperative complications and 6-months mortality was 3.9% (n=25). Grip strength, gait speed, MFS and ASA classification could predict postoperative complication but only MFS (Hazard Ratio = 1.564, 95% CI, 1.283-1.905, p < 0.001) could predict 6-months mortality after full adjustment. MFS (C index = 0.747) had superior prognostic utility than age (0.638, p value = 0.008), grip strength (0.566, p value < 0.001) and ASA classification (0.649, p value = 0.004). MFS only had additive predictive value on both age (C-index of 0.638 (age) vs 0.754 (age +MFS), p = 0.001) and ASA classification (C index of 0.649 (ASA) to 0.762 (ASA + MFS), p < 0.001) for postoperative complication, but gait speed or grip strength had no statistical additive prognostic value on both age and ASA classification.

